# 

**Published:** 1857-06

**Authors:** 


### INDEX TO VOLUME THIRTEENTH.

For original communications, look for the names of the authors as well as the titles of the subjects.

For selected and editorial articles, look for the titles of the subjects only.

For notices of books, look for names of authors.

### ILLUSTRATIONS.

PAGE.
Fibro-plastic Cells and Nuclei, ....................................709
Glandular Tube of Parotid,.........................................711
Epithelium of Glandular Tube, .....................................711

### A.

PAGE.
Address of Dr. Willard, ............ 703
Albuminuria Independent of Organic Disease of the Kidneys. By James M. Newman, M. D., ........ 589
Albuminuria, Connection of, with the Development of Puerperal Convulsions. By Jas. M. Newman, M. D. (Buff. Med. Assoc.),.............. 712
Alcoholic Treatment of Tuberculosis. By Thos. Cushing,............... 532
American Med. Association, Meeting of,........................ 31
" " Transactions of, 492
Amylene. By John G. Orton, M.D., 167
Aneurismal Tumor, treated by a New Method,......................... 418
Annual Dinner of the Erie Co. Medical Society, .................... 571
Apoplexy and Paralysis, ........... 205
Arthritis Chronic Rheumatic, Specimen of, (Buff. Med. Assoc.)...... 349
Asthma, Cases of,.................. 1

### B.

Bartlett on the Fevers of the United States,........................ 224
PAGE.
Biegler, Augustus P., Trial of, for Infanticide, ........................ 12
Bile, Constitution and Physiology of, By Prof. J. C. Dalton, Jr.,....365, 381
Bleeding from the Ear in consequence of an Injury to the Chin, ......... 433
Blood Poisoning. By J. T. Jamieson, M. D.,..................... 462
Borden's Condensed Milk,.......... 421
Bright's Disease, (Buff. Med. Assoc.) 151
" " .................. 527
Bronchitis, Plastic or Pseudo-membranous, Case of,................ 1
Braithwaite's Retrospect, and Ayer's Cherry Pectoral and Cathartic Pills,............................ 247
Buffalo Med. Association, Abstract of Proceedings of,................ 149
" " ................ 216
" " ................ 268
" " ................ 346
" " ................ 403
" " ................ 536
" " ................ 665
" " ................ 712

C. PAGE.

Cancer, Colloid, (Buff. Med. Assoc.), 149
Cavities, Pulmonary, Diagnostic value of Symptoms in, .......... 50
Cataract, Cases and Operations,.... 163
Cazeaux, Midwifery, ............... 547
Cents of Copper and Nickel, Discussion of, (Buff. Med. Assoc.),...... 270
Churchill on Diseases of Females,... 169
Cushing, Thomas. Alcoholic Treatment of Tuberculosis,............ 532

D.

Death of Charlotte Brontë,.......... 416
Death of Dr. Morgan G. Lewis....... 631
Deaths, Monthly Record of, (April,) 45
" " " (May,) 103
" " " (June,) 183
" " " (July,) 224
" " " (August,) 302
" " " (September,) 363
" " " (October,) 414
" " " (November,) 501
" " " (December,) 553
" " " (January,) 619
" " " (February,) 693
" " " (March,) 759
Diagnosis of Cancer, Discussion in the French Academy of Medicine. Translated by C. B. Hutchins, M. D., ........................ 13
Dislocation of Elbow, Simple Mode of Reduction of, ................ 52
Dislocation of Radius and Ulna Outward, Ancient,.................. 684
Dislocation of Hip-Joint, Reduction by Manipulation, Fragments of the Literature. Compiled by Prof. F. H. Hamilton, M. D.,.....325, 514, 581
Dislocation of the Femur upon the Dorsum Ilii, Reduction by Manipulation. By Prof. Frank H. Hamilton, M. D., .................... 682
Dissection Wounds. By E. R. Maxson, M. D., ...................... 472
Dissection Wounds. Report by J. F. Miner, M. D., (Buff. Med. Assoc.,) 478
Dissec'n Wounds. (Buff. Med. Ass.) 153
Duties of State Assayers in Relation to Quack Medicines, ............ 417

E. PAGE.

Ecstacy, Case of, ................ 139
Embalment,....................... 188
Empyema, Case of,................ 1
Empyema, Treated by Injection of Iodine, ........................ 429
Encephaloid Disease of the Antrum, in the Practice of Prof. F. H. Hamilton, M. D. Reported by B. H. Lemon, M. D.,................. 355
Erie Co. Medical Society, Annual Meeting of,...................... 628
Erysipelas, Muriated Tinct. of Iron taken internally by mistake, (Buff. Med. Assoc.),................. 682
Eve, Prof. Paul F., Collection of Remarkable Cases in Surgery,...... 381
Experiments on Chickens, Bending of Bones, (Buff. Med. Assoc.), .... 350

F.

Fat Men, ......................... 314
Female Doctors, .................. 191
Fell, the American Cancer Doctor,.. 313
Fever, Pueperal, New York Academy of Medicine, ................105, 226
Fever, Typhoid vs. Remittent,...... 59
Fever, Typhoid, Case, with Spasmodic Respiration,................ 398
Fever, Intermittent,.............. 400
Flint, Austin, Jr., M. D., Microscopic Examination of Tumor of Parotid, 707
Fractures. By Prof. F. H. Hamilton, M. D., Superior Maxilla, Ethmoid, and other bones of the Face, ..... 257
" Zygoma & Zygomatic Arch,. 321
" Inferior Maxilla, ........385, 449
" Sternum, ................. 520
" Ribs and their Cartilages,... 578
Fracture, Inferior Maxilla. By O. C. Gibbs, M. D., .................. 526
Fracture, Neck of Femur within the Capsule, (Buff. Med. Assoc.), .... 151
Fracture, Neck of Femur, with complete Bony Union, (Buff. Med. Assoc.),...................... 269
Fracture of the Os Frontis. By B. H. Lemon, M. D., (Buff. Med. Assoc.) 286

G.

Gastralgia and Enteralgia, .......... 477

PAGE.
Gibbs, O. C., M, D., Case of Morbus Coxarius, .................... 201
Gibbs, Fracture of Inferior Maxilla,. 526
Gonorrhœa, with Epididymitis, Case, 100
Gonorrhœa, with Testitis, Case of,... 101
Green, Horace, M. D., Lesions of the Epiglottic Cartilage, ............ 382
Gross, Prof., Elements of Pathological Anatomy,.................. 218
Growths from the Interior of Uterus, (Buff. Med. Assoc.),............. 665

H.

Hamilton, Prof. F. H., Fracture of Os
29 Hyoides........................ ~~130~~
Hamilton, of Fracture Malar Bones, Superior Maxillary, Ethmoid and other Bones of the Face,.......... 257
Hamilton, Fracture of Zygoma and Zygomatic Arch,............... 321
Hamilton, Fracture of Inferior Maxilla,.........................385, 449
Hamilton, Fracture of the Sternum,. 520
Hamilton, Fracture of the Ribs and their Cartilages,................ 578
Hamilton, Dislocation of Femur upon Dorsum Ilii, Reduction by Manipulation,....................... 682
Hamilton, Tumor of Parotid Gland, Extirpation of the entire Gland, .. 705
Hamilton, Reduction of Dislocation of Hip-Bone by Manipulation, Fragments of the Literature of the Method,................325, 514, 581
Hard Hit,........................ 62
Heart, Disease of Valves, Specimen of, (Buff. Med. Assoc.), ........... 488
Health Physician's Annual Report,. 699
Hernia, Strangulated Femoral, Operation,.......................... 96
Honors Conferred upon American Physicians, ....................... 509
Hospital, Buffalo Charity, Cases occurring in the Med. Ward. Treated by Prof. Flint. Reported by A. W. Nichols, M. D.,............ 1
" " ................ 65
" " ................ 139
" " ................ 205
" " ................ 291
Hospital, Buffalo Charity,.......... 337
" " ................ 398
" " ................ 475
" " ................ 527
" " ................ 608
" " ................ 641
Hospital, Buffalo Charity. Surgical Reports by B. H. Lemon, M. D., .. 95
" " ................ 163
" " ................ 211
" " ................ 351
Hospital, Buffalo General,.......... 127
" " ................ 512
Hutchins, C. B., M. D., Translation of the Discussion on the Diagnosis of Cancer, in the French Academy of Medicine, ...................... 73
Hydrophobia, Case of, (Buff. Med. Assoc.),......................... 675

I.

Imperforate Rectum, Operation successful, (Buff. Med. Assoc.),...... 544
Insanity, Feigned, Detection of, .... 48
Influence of Marriages of Consanguinity upon Offspring, ....... 249
Injury to Forearm, Case of, (Buff. Med. Assoc.),................. 403
Isaacs, Structure and Physiology of the Kidney,..................... 221

J.

Jamieson, J. T., M. D., Pleuro-Pneumonia,......................... 197
Jamieson, Blood Poisoning, ........ 402
Jenner Monument,................. 251
July Number,...................... 127

L.

Lemon, B. H., M. D., Case of Meningo-Cerebritis occurring in the Practice of Dr. Wilcox, ............. 193

M.

Maxson, E. R., M. D., on Dissection Wounds,....................... 472
Marshall Hall, Obituary of,........ 301
McClintock, Dr. James,............ 128
Medical Society of County of Erie, Semi-annual Meeting of,.......... 121

PAGE.
Medical Society of South-Western New York, Constitution of, ...... 127
Med. Society of South Western New York, Meeting of, .............. 127
Medical Spiritualism, ............. 191
Medication of Respiratory Passages, 426
Medico-Legal Insanity, ........... 122
Meacham, Wm. G., M. D., Case of Urticaria, ........................ 297
Meningo-Cerebritis, Case in Practice of Dr. Wilcox. Reported by B. H. Lemon, M. D., ................. 193
Mendenhall's Medical Student's Vade Mecum, ...................... 551
Microscopical Examination of Spots of Blood, by M. Robin, .......... 555
Microscopical Examination of Tumor of Parotid Gland. By A. Flint, Jr., M. D., ........................ 767
Miller, Principles and Practice of Obstetrics, ..................... 616
Morbus Coxarius, Case of. By O. G. Gibbs, M. D., ................. 201
More Tinkering, ................. 60
Mucous Membrane of the Uterus, Specimens of Exfoliation of, (Buff. Med. Assoc.), ..................... 539

N.

New Volume, ..................... 53
Neuralgia, Intercostal, ...........65, 401
New Stand for the Compound Microscope, ........................ 637
Newman, James M., M. D., Albuminuria Independent of Organic Disease of the Kidney, .............. 589
Newman, Connection of Albuminuria with the Development of Puerperal Convulsions, and the employment of Chloroform as a Remedial Measure, (Buff. Med. Assoc.), ......... 712
New Year and Medical Journals,... 509
New York State Med. Society and Medical Fees, ................. 242
Niles, Addison, M. D., Chronic Pleuritis, ........................ 330
Nott and Gliddon. Indigenious Races of the Earth, ............. 90

O.

Opening of Medical Schools, ........ 440

PAGE.
Orton, John G., M. D., Amylene,.... 167
Os Hyoides, Fracture of. By F. H. Hamilton, M. D., ................ 130
Ovarian Tumor, Specimen of, (Buff. Med. Assoc.), .................. 536

P.

Parotid Gland, Complete Extirpation of. By Prof. F. H. Hamilton,.... 705
Pathological Society of Philadelphia, 704
Paracentesis Thoracis, ............ 252
Paralysis, ........................ 608
Peaslie's Human Histology, ........ 405
Pepsin, Physiological and Chemical Properties, ....................... 434
Perforation of Appendix Vermiformis (Buff. Med. Assoc.), ............. 280
Pleuralgia, ....................... 65
Pleuro Pneumonia. By J. T. Jamieson, M. D., ....................... 197
Pleuritis, Chronic. By Addison Niles, M. D., ..................... 330
Pneumonitis, Acute Lobar, ......... 291
Pueumonitis, Acute Lobar. Treatment by Opiates and Stimulants, . 337
Powers of County Med. Societies, 151, 185
Polypus of the Uterus, (Buff. Med. Assoc.), ........................ 150
Poisoning by Syrup of Poppies and Belladonna, (Buff. Med. Assoc.),.. 281
Prostration and Coma, the effects of Fatigue, and Exposure to the Heat of the Sun, (Buff. Med. Assoc.)... 346
Professional Honor, ............... 442
Prurigo, .......................... 400
Proceedings of the Physicians' Meeting at Cleveland, ................ 701
Pseudo-Apoplexy, Arcus Senilis,... 205
Ptosis, Case of, Operation, ........ 211
Pulmonary Tuberculosis, .......... 641
Pulmonary Tuberculosis, Bulbous Enlargement of Fingers and Toes,. 475
Pulmonary Tuberculosis, with Sub-Acute Rheumatism, ............... 477
Puerperal Convulsions, Case of, (Buff. Med. Assoc.) .................. 679
Pyæmia, Case of, (Buff. Med. Assoc.) 667

Q.

Quarantine Convention, Philadelphia, May, 1857, ................. 115

### R.

PAGE.
Reproduction of Bones and Joints after their Removal in cases of Whitlow, ..................... 572
Respiratory Muscle,.................. 424
Rectal Hæmorrhage, (Buff. Med. Association,)..................... 279
Revaccination, Experiments in, .... 47
Rheumatism, (Buff. Med. Assoc.)... 216
Rigby on Female Diseases,.......... 38
Robin, Dr. Ch., of Paris,............ 313
Rupture of the Gall-Bladder,........ 427

### S.

Sale of Poisons, .................. 634
Scales of Bone from the Falx Cerebri, (Buff. Med. Assoc.) .............. 539
Sorghum, or Chinese Sugar Cane, 504, 583
Strictures, Intractible, Beneficial effects of openiug the Urethra behind them,............................ 51
Stricture, Impassible, Operation,.... 352
Surgical Reports, .................... 128
Supra-Renal Capsules, .............. 621
Syphilization,.......................... 54
Syphilis, Secondary and Tertiary, Pseudo-membranous Croup, Death, 98
PAGE.
Synovitis, Sub-acute, Case of,....... 212
Synovitis, with Ulceration of the Cartilages, ............................ 214
Sydenham Society,.............503, 635

### T.

Thoracentesis, Two cases of,......... 49
Transactions of the N. Y. State Med. Society, New Hampshire Medical Society, and Penn. State Medical Society,.......................... 356

### U.

University of Buffalo,......190, 315, 695
Urticaria, Case of. By William G. Meacham, M. D.,................. 297

### V.

Vesico-Vaginal Fistulâ, Case of,.... 351

### W.

West, Lectures of Diseases of Women, Part I,....................... 685
Whisky in Tuberculosis, (Buff. Med. Assoc.) ......................... 153

*American Pharmaceutical Association.*—The next annual meeting of this association will be held in Philadelphia, on the 8th of September next.

